# Association between tea consumption and risk of cancer: a prospective cohort study of 0.5 million Chinese adults

**DOI:** 10.1007/s10654-019-00530-5

**Published:** 2019-05-31

**Authors:** Xinyi Li, Canqing Yu, Yu Guo, Zheng Bian, Zewei Shen, Ling Yang, Yiping Chen, Yongyue Wei, Hao Zhang, Zhe Qiu, Junshi Chen, Feng Chen, Zhengming Chen, Jun Lv, Liming Li, the China Kadoorie Biobank Collaborative Group, Junshi Chen, Zhengming Chen, Robert Clarke, Rory Collins, Yu Guo, Liming Li, Jun Lv, Richard Peto, Robin Walters, Daniel Avery, Ruth Boxall, Derrick Bennett, Yumei Chang, Yiping Chen, Zhengming Chen, Robert Clarke, Huaidong Du, Simon Gilbert, Alex Hacker, Mike Hill, Michael Holmes, Andri Iona, Christiana Kartsonaki, Rene Kerosi, Ling Kong, Om Kurmi, Garry Lancaster, Sarah Lewington, Kuang Lin, John McDonnell, Iona Millwood, Qunhua Nie, Jayakrishnan Radhakrishnan, Paul Ryder, Sam Sansome, Dan Schmidt, Paul Sherliker, Rajani Sohoni, Becky Stevens, Iain Turnbull, Robin Walters, Jenny Wang, Lin Wang, Neil Wright, Ling Yang, Xiaoming Yang, Zheng Bian, Yu Guo, Xiao Han, Can Hou, Jun Lv, Pei Pei, Chao Liu, Yunlong Tan, Canqing Yu, Zengchang Pang, Ruqin Gao, Shanpeng Li, Shaojie Wang, Yongmei Liu, Ranran Du, Yajing Zang, Liang Cheng, Xiaocao Tian, Hua Zhang, Yaoming Zhai, Feng Ning, Xiaohui Sun, Feifei Li, Silu Lv, Junzheng Wang, Wei Hou, Mingyuan Zeng, Ge Jiang, Xue Zhou, Liqiu Yang, Hui He, Bo Yu, Yanjie Li, Qinai Xu, Quan Kang, Ziyan Guo, Dan Wang, Ximin Hu, Jinyan Chen, Yan Fu, Zhenwang Fu, Xiaohuan Wang, Min Weng, Zhendong Guo, Shukuan Wu, Yilei Li, Huimei Li, Zhifang Fu, Ming Wu, Yonglin Zhou, Jinyi Zhou, Ran Tao, Jie Yang, Jian Su, Fang liu, Jun Zhang, Yihe Hu, Yan Lu, Liangcai Ma, Aiyu Tang, Shuo Zhang, Jianrong Jin, Jingchao Liu, Zhenzhu Tang, Naying Chen, Ying Huang, Mingqiang Li, Jinhuai Meng, Rong Pan, Qilian Jiang, Jian Lan, Yun Liu, Liuping Wei, Liyuan Zhou, Ningyu Chen, Ping Wang, Fanwen Meng, Yulu Qin, Sisi Wang, Xianping Wu, Ningmei Zhang, Xiaofang Chen, Weiwei Zhou, Guojin Luo, Jianguo Li, Xiaofang Chen, Xunfu Zhong, Jiaqiu Liu, Qiang Sun, Pengfei Ge, Xiaolan Ren, Caixia Dong, Hui Zhang, Enke Mao, Xiaoping Wang, Tao Wang, Xi Zhang, Ding Zhang, Gang Zhou, Shixian Feng, Liang Chang, Lei Fan, Yulian Gao, Tianyou He, Huarong Sun, Pan He, Chen Hu, Xukui Zhang, Huifang Wu, Pan He, Min Yu, Ruying Hu, Hao Wang, Yijian Qian, Chunmei Wang, Kaixu Xie, Lingli Chen, Yidan Zhang, Dongxia Pan, Qijun Gu, Yuelong Huang, Biyun Chen, Li Yin, Huilin Liu, Zhongxi Fu, Qiaohua Xu, Xin Xu, Hao Zhang, Huajun Long, Xianzhi Li, Libo Zhang, Zhe Qiu

**Affiliations:** 1 Department of Epidemiology and Biostatistics, School of Public Health, Peking University Health Science Center, 38 Xueyuan Road, Beijing, 100191 China; 2 Department of Epidemiology, Harvard T.H. Chan School of Public Health, Boston, MA 02115 USA; 3 Chinese Academy of Medical Sciences, Beijing, China; 4 Clinical Trial Service Unit and Epidemiological Studies Unit (CTSU), Nuffield Department of Population Health, University of Oxford, Oxford, UK; 5 Department of Biostatistics, School of Public Health, Nanjing Medical University, Nanjing, China; 6 Liuyang Center for Disease Control and Prevention, Liuyang, Hunan China; 7 China National Center for Food Safety Risk Assessment, Beijing, China; 8 Key Laboratory of Molecular Cardiovascular Sciences (Peking University), Ministry of Education, Beijing, China; 9 Peking University Institute of Environmental Medicine, Beijing, China

**Keywords:** Tea consumption, Cancer, Lung cancer, Stomach cancer, Colorectal cancer, Liver cancer, Breast cancer, Cervix uteri cancer, Prospective cohort study

## Abstract

Current experimental and epidemiological studies provide inconsistent evidence toward the association between tea consumption and cancer incidence. We investigated whether tea consumption was associated with the incidence of all cancers and six leading types of cancer (lung cancer, stomach cancer, colorectal cancer, liver cancer, female breast cancer and cervix uteri cancer) among 455,981 participants aged 30–79 years in the prospective cohort China Kadoorie Biobank. Tea consumption was assessed at baseline (2004–2008) with an interviewer-administered questionnaire. Cancer cases were identified by linkage to the national health insurance system. Cox proportional hazard regression models were used to estimate hazard ratios (HRs) and 95% confidence intervals (CIs). In the present population, daily tea consumers were more likely to be current smokers and daily alcohol consumers. 22,652 incident cancers occurred during 10.1 years follow-up (5.04 cases/1000 person-years). When we restricted analyses to non-smokers and non-excessive alcohol consumers to minimize confounding, tea consumption was not associated with all cancers (daily consumers who added tea leaves > 4.0 g/day vs. less-than-weekly consumers: HR, 1.03; 95%CI, 0.93–1.13), lung cancer (HR, 1.08; CI, 0.84–1.40), colorectal cancer (HR, 1.08; CI, 0.81–1.45) and liver cancer (HR, 1.08; CI, 0.75–1.55), yet might be associated with increased risk of stomach cancer (HR, 1.46; CI, 1.07–1.99). In both less-than-daily and daily tea consumers, all cancer risk increased with the amount of tobacco smoked or alcohol consumed. Our findings suggest tea consumption may not provide preventive effect against cancer incidence.

**Electronic supplementary material:**

The online version of this article (10.1007/s10654-019-00530-5) contains supplementary material, which is available to authorized users.

## Introduction

Tea is a commonly consumed beverage in various populations. In vitro and in vivo experiments have demonstrated that tea polyphenols may prevent the occurrence of cancer through antioxidation, growth inhibition and apoptosis induction [1]. However, the association between tea consumption and cancer risk remains controversial based on evidence from human studies. Case–control studies consist of the majority of evidence supporting the cancer-preventive effects of tea, while they are subject to recall bias and reverse causality as prediagnostic symptoms of cancer may change the drinking habits. On the other hand, most recent prospective cohort studies have not found associations between tea consumption and cancer incidence [2–5]. Some studies even suggested elevated risks for stomach cancer and several subtypes of breast cancer among tea consumers, probably because the caffeine in tea could stimulate secretion of gastric acid and increase the level of estrogen and progesterone [6–8].

Despite lack of solid evidence, it is widely believed among Chinese that tea consumption is beneficial for cancer prevention. Some health channels in mass media even specifically suggest that drinking tea can help smokers alleviate the harm of tobacco use. This common notion, together with other cultural and social factors, contributes to the clustering habits of smoking, alcohol and tea consumption among Chinese men. However, most of the prospective studies investigating tea consumption and cancer risk were conducted in Europe, the U.S., and Japan where no such clustering of lifestyle risk factors was evident in tea consumers [5, 9–12].

The China Kadoorie Biobank (CKB) is a large prospective cohort including 0.5 million adults from both urban and rural regions geographically spread across China. In previous study of this population, we have observed increased esophageal cancer risk among hot tea consumers who either smoked or consumed alcohol excessively [13]. In the current study, we further examined the association between tea consumption and risks of all cancers as well as the other six leading cancers in our population—lung cancer, stomach cancer, colorectal cancer, liver cancer, and female breast cancer and cervix uteri cancer. The large sample size and adequate cancer cases enabled us to evaluate the association among people who neither smoked nor drank alcohol excessively and to investigate whether tea consumption could offer any benefits to those with an unhealthy lifestyle.

## Methods

### Study population

Detailed descriptions of the CKB study design and characteristics of the study participants have been given elsewhere [14, 15]. In brief, a total of 512,715 participants aged 30–79 years were recruited between 2004 and 2008 from five urban and five rural regions of China. Each eligible participant completed a written informed consent, an interviewer-administered electronic questionnaire, physical measurements, and a blood draw. The Ethical Review Committee of the Chinese Center for Disease Control and Prevention (Beijing, China) and the Oxford Tropical Research Ethics Committee, University of Oxford (UK) approved the study.

In the present analysis, we excluded participants with previously diagnosed cancer (n = 2578) or missing data for body mass index at baseline (n = 2), or who were lost to follow-up shortly after baseline (n = 1). Former smokers (n = 30,561) who had stopped smoking for ≥ 6 months, and participants whose consumption frequency of alcohol (n = 20,952) or tea (n = 11,576) had reduced from at least weekly to less than weekly at baseline were also excluded to avoid potential reverse causality. The final analyses included 455,981 participants. In the analyses of breast cancer and cervix uteri cancer, we further excluded female participants with a history of lumpectomy (n = 2333) and hysterectomy (n = 11,215), respectively.

### Assessment of tea consumption

In the baseline questionnaire, we asked participants to report their frequency of tea consumption (never, only occasionally, only at certain seasons, monthly but less than weekly, or at least once a week) during the past 12 months. Less-than-weekly consumers were asked whether they ever had consumed tea weekly for at least 1 year. Weekly consumers were asked about their days consuming in a typical week (1–2, 3–5, or 6–7 days), cups of tea (in 300 ml-sized cups) in one drinking day, tea leaves (in grams) added each time, times of changing tea leaves in one drinking day, type of tea consumed most commonly (green tea, oolong tea, black tea, or others), preferred temperature at which tea was consumed (room temperature/warm, hot, or burning hot), and age since they started drinking tea weekly. Participants were provided with a pictorial guide to gauge the amount of tea in cups and tea leaves in grams. The tea leaves added in one drinking day were calculated by multiplying the amount added each time by times of changing tea leaves. The Spearman’s correlation coefficient between the baseline survey and a subsequent re-survey at a mean interval of 2.6 years among 4405 weekly tea consumers was 0.63 for tea leaves added and 0.53 for cups of tea consumed [13].

For the present analyses, all participants were categorized into three groups regarding their consumption frequency: less than weekly, weekly, and daily. Daily tea consumers were further categorized according to tea leaves added per day: 0.1–2.0, 2.1–4.0 and > 4.0 g.

### Assessment of tobacco smoking, alcohol consumption, and other covariates

We asked ever smokers about their frequency, type, and the amount of tobacco (in cigarette or equivalent amount of tobacco; 1 cigarette = 1 g of tobacco = 0.5 cigars [16]) smoked per day, and time since they quitted for former smokers. Participants who quitted smoking for less than 6 months were still regarded as current smokers and included in the present analyses. We asked participants who drank alcohol at least once a week to report their typical consumption frequency, type of alcoholic beverage consumed habitually, and the amount of alcohol consumed on a typical drinking day. We used this information to calculate pure alcohol intake in grams on a typical drinking day [17]. We also asked less-than-weekly consumers whether they ever had consumed alcohol weekly for at least a year.

Other covariates obtained from the baseline questionnaire included socio-demographic characteristics (age, sex, education, occupation, marital status, and household income), lifestyle behaviors (physical activity, and intakes of red meat, fresh fruits and vegetables, and preserved vegetables), personal medical history (diabetes, heart disease, stroke, peptic ulcer, chronic hepatitis or cirrhosis, and gallstone or gallbladder disease), reproductive history (menopausal status, age at first period, ever using oral contraceptives, number of live births), secondhand smoke exposure, and family history of cancer. Trained staff measured weight, height, and circumferences of waist and hip using calibrated instruments, as well as doing blood tests for hepatitis B surface antigen (HBsAg) and blood glucose. Prevalent diabetes was defined as measured fasting blood glucose ≥ 7.0 mmol/l, measured random blood glucose ≥ 11.1 mmol/l, or self-reported prior diagnosis of diabetes at baseline.

### Ascertainment of incident cancer

The vital status of all CKB participants was obtained through linkage with local disease surveillance points system death registries and residential records. Information on cancer incidence was collected through linkage with established disease registries, electronic linkage with the national health insurance claim database. Participants who failed to be linked to local health insurance database were actively followed annually by staff to ascertain their status including hospital admission and moving out of the study area. Trained staff blinded to baseline information coded all diagnoses using the International Classification of Diseases, Tenth Revision (ICD-10). The outcomes of the present analysis were all cancers (C00–C97), stomach cancer (C16), colorectal cancer (C18–C20), liver cancer (C22), lung cancer (C33–C34), and female breast cancer (C50) and cervix uteri cancer (C53).

### Statistical analysis

We calculated person-years at risk from the baseline to the first cancer diagnosis, death, loss to follow-up or 31 December 2016, whichever occurred first. In the cancer site-specific analyses, participants with more than one cancer diagnosis during the follow-up period were censored at the first cancer diagnosis. We used a multivariable Cox proportional hazards model to estimate the hazard ratio (HR) and 95% confidence interval (CI), with age as the underlying time scale, and stratified by ten study regions and age at baseline in the 5-year interval. The proportional hazards assumption for the Cox model was checked using Schoenfeld residuals, and no violation was found.

Multivariable models were adjusted for sex (for whole cohort), education, occupation, marital status, household income, tobacco smoking, alcohol consumption, physical activity, intakes of red meat, fresh fruits and vegetables, body mass index, waist-hip-ratio, prevalent diabetes, family history of cancer, menopausal status (for women only). Additional adjustments for secondhand smoke exposure were done in analysis of lung cancer; and intake of preserved vegetables, in analysis of stomach cancer; HBsAg status, in liver cancer analysis; age at first period, history of using oral contraceptives, number of live births, in breast cancer analysis. We tested linear trend of cancer risk across different groups of tea consumption among daily tea consumers by assigning the median of tea leaves added to three categories.

To more strictly control for confounding caused by smoking and alcohol consumption, we restricted the analyses to participants who neither smoked nor consumed alcohol excessively. Such an association between tea consumption and cancer risk was also examined in their less healthy counterpart. Excessive alcohol consumption was defined as daily pure alcohol intake of 15 grams or more, while non-excessive consumption was defined as less-than-daily intake or daily pure alcohol intake less than 15 grams. We constructed the cross-product term between tea consumption category and indicator for smoke or excessive alcohol consumption (either smoke or consume alcohol excessively, neither smoke nor consume alcohol excessively), and tested multiplicative interaction by using likelihood ratio test comparing models with and without the cross-product term. We also examined the association between tobacco smoking and cancer risk, and between alcohol consumption and cancer risk by different status of tea consumption.

In the sensitivity analyses, we excluded participants whose outcome occurred during the first 3 years of follow-up to avoid reverse causality. We additionally adjusted for history of peptic ulcer in analyses of stomach cancer, and history of chronic hepatitis or cirrhosis and gallstone or gallbladder disease in analyses of liver cancer.

We performed all analyses with Stata version 14.2 (StataCorp, TX, USA).

## Results

Participants included in analyses had a mean age of 50.9 ± 10.5 years. Of 455,981 participants, 36.1% were men; 56.1% resided in rural areas; and 42.1% of men and 16.1% of women reported consuming tea almost every day. Daily tea consumers were more likely to be current smokers and daily alcohol consumers. The amount of tobacco or alcohol consumption also increased with tea leaves added in daily tea consumers (Table 1). More baseline characteristics across tea consumption categories were presented in Appendix Table 1.

**Table 1:** Baseline characteristics of 455,981 participants according to tea consumption

	Less than weekly	Weekly	Daily			*P*_*trend*_
			≤ 2.0 g	2.1–4.0 g	>4.0 g	
No. of participants, n (%)^a^	307,057 (67.3)	32,722 (7.2)	44,988 (9.9)	41,621 (9.1)	29,593 (6.5)	< 0.001
Men, %	25.6	51.4	48.8	59.4	76.2	< 0.001
Urban area, %	44.7	50.0	35.3	37.9	50.9	< 0.001
Age, years	50.9	48.8	52.0	51.7	50.7	< 0.001
Tobacco smoking						
Current smokers, %						
Men	59.8	72.2	76.2	79.6	85.7	< 0.001
Women	2.0	3.0	3.5	4.3	6.2	< 0.001
Amount smoked per day, cigarette or equivalent tobacco						
Men	17.3	17.3	17.5	18.2	20.9	< 0.001
Women	9.5	9.8	10.9	10.7	12.9	< 0.001
Duration of smoking, years						
Men	28.3	27.6	27.7	28.1	28.9	< 0.001
Women	30.6	28.6	29.6	29.1	28.2	< 0.001
Alcohol consumption						
Daily consumer, %						
Men	16.8	20.5	26.5	27.8	29.8	< 0.001
Women	0.7	1.6	2.3	2.6	3.9	< 0.001
Amount consumed per day, g of pure alcohol						
Men	57.5	59.4	58.4	60.7	65.5	< 0.001
Women	28.0	27.8	29.5	30.8	31.2	0.024
Duration of consumption, years						
Men	22.2	21.7	22.1	21.9	22.4	0.152
Women	14.5	14.2	15.2	15.2	16.3	0.011
Tea consumption						
Tea leaves added per day, g	–	–	1.8	3.5	8.9	< 0.001
Tea consumed per day, cups^b^	–	–	3.3	4.4	6.3	< 0.001
Green tea consumer, %	–	85.1	85.7	85.7	85.3	0.065
Preferred hot or burning hot tea, %		54.2	56.1	60.2	71.2	< 0.001
Age starting regular consumption, years	–	29.7	28.4	27.7	26.0	< 0.001
Duration of consumption, years	–	21.3	22.5	23.3	25.0	< 0.001

The results are presented as adjusted means or percentages, with adjustment for age, sex, and study area, as appropriate

^a^The numbers in parentheses indicate the proportion of participants in five tea consumption categories

^b^1 cup = 300 ml

### Tea consumption and cancer risk

During a median follow-up of 10.1 years (4.5 million person-years), we documented 22,652 cancer cases (5.04 cases/1000 person-years) which were first diagnosed, including 4046 (17.9%) lung cancers, 2366 (10.4%) stomach cancers, 2267 (10.0%) colorectal cancers, 1874 (8.3%) liver cancers, 1984 (8.8%) female breast cancers and 868 (3.8%) cervix uteri cancers. In the multivariable-adjusted models, a larger amount of tea leaves consumption was associated with higher risk of all cancers, lung cancer, and stomach cancer among all participants (Table 2; *P*_*trend*_< 0.001). A clear attenuation of the association was observed after tobacco smoking were adjusted. After adjustment for all potential confounders, daily consumers who added tea leaves more than 4.0 g/day showed elevated risk of all cancers (HR, 1.13; CI, 1.07–1.20), lung cancer (HR, 1.31; CI, 1.17–1.46), and stomach cancer (HR, 1.18; CI, 1.02–1.37), compared with less-than-weekly consumers. Tea consumption was not associated with risk of colorectal cancer, liver cancer, female breast cancer and cervix uteri cancer.

**Table 2:** HRs (95% CIs) for the association between tea consumption and cancer risk among 455,981 participants

	Less than weekly	Weekly	Daily			*P*_*trend*_^f^
			≤ 2.0 g	2.1–4.0 g	> 4.0 g	
All cancers						
Cases	14,472	1493	2441	2329	1917	
Cases/PYs (/1000)	4.78	4.60	5.56	5.71	6.54	
Sex-adjusted	1.00	1.06 (1.01, 1.12)	1.09 (1.04, 1.14)	1.14 (1.09, 1.20)	1.26 (1.20, 1.33)	< 0.001
Multivariable-adjusted	1.00	1.07 (1.01, 1.13)	1.09 (1.04, 1.14)	1.15 (1.09, 1.20)	1.26 (1.20, 1.33)	< 0.001
Further adjusted for tobacco smoking	1.00	1.04 (0.99, 1.10)	1.06 (1.01, 1.11)	1.09 (1.04, 1.15)	1.16 (1.10, 1.23)	< 0.001
Further adjusted for alcohol consumption	1.00	1.04 (0.98, 1.10)	1.03 (0.98, 1.08)	1.07 (1.02, 1.12)	1.13 (1.07, 1.20)	< 0.001
Lung cancer^a^						
Cases	2278	248	503	513	504	
Cases/PYs (/1000)	0.74	0.76	1.13	1.25	1.70	
Sex-adjusted	1.00	0.97 (0.85, 1.11)	1.11 (1.00, 1.24)	1.22 (1.10, 1.36)	1.60 (1.43, 1.77)	< 0.001
Multivariable-adjusted	1.00	1.00 (0.87, 1.15)	1.12 (1.01, 1.25)	1.25 (1.12, 1.39)	1.62 (1.45, 1.80)	< 0.001
Further adjusted for tobacco smoking	1.00	0.94 (0.82, 1.08)	1.03 (0.93, 1.15)	1.11 (0.99, 1.23)	1.32 (1.18, 1.47)	0.001
Further adjusted for alcohol consumption	1.00	0.95 (0.83, 1.08)	1.03 (0.92, 1.14)	1.10 (0.99, 1.22)	1.31 (1.17, 1.46)	0.001
Stomach cancer^b^						
Cases	1456	173	239	221	277	
Cases/PYs (/1000)	0.48	0.53	0.54	0.54	0.94	
Sex-adjusted	1.00	1.09 (0.93, 1.29)	0.90 (0.78, 1.05)	0.88 (0.76, 1.03)	1.27 (1.10, 1.46)	< 0.001
Multivariable-adjusted	1.00	1.12 (0.95, 1.32)	0.93 (0.80, 1.07)	0.92 (0.79, 1.07)	1.29 (1.12, 1.49)	< 0.001
Further adjusted for tobacco smoking	1.00	1.09 (0.92, 1.28)	0.89 (0.77, 1.03)	0.87 (0.74, 1.02)	1.20 (1.03, 1.39)	< 0.001
Further adjusted for alcohol consumption	1.00	1.08 (0.92, 1.28)	0.88 (0.76, 1.02)	0.86 (0.73, 1.00)	1.18 (1.02, 1.37)	< 0.001
Colorectal cancer						
Cases	1431	169	254	216	197	
Cases/PYs (/1000)	0.47	0.52	0.57	0.52	0.66	
Sex-adjusted	1.00	1.20 (1.01, 1.41)	1.11 (0.96, 1.28)	1.06 (0.91, 1.24)	1.23 (1.04, 1.44)	0.108
Multivariable-adjusted	1.00	1.17 (0.99, 1.38)	1.09 (0.94, 1.26)	1.03 (0.88, 1.21)	1.19 (1.01, 1.40)	0.146
Further adjusted for tobacco smoking	1.00	1.16 (0.99, 1.37)	1.08 (0.93, 1.25)	1.02 (0.87, 1.19)	1.16 (0.98, 1.37)	0.213
Further adjusted for alcohol consumption	1.00	1.15 (0.97, 1.36)	1.05 (0.91, 1.22)	1.00 (0.85, 1.17)	1.13 (0.96, 1.33)	0.265
Liver cancer^c^						
Cases	1143	138	216	223	154	
Cases/PYs (/1000)	0.37	0.42	0.49	0.54	0.52	
Sex-adjusted	1.00	0.98 (0.82, 1.18)	0.96 (0.82, 1.13)	1.00 (0.85, 1.17)	0.95 (0.79, 1.13)	0.794
Multivariable-adjusted	1.00	1.00 (0.83, 1.21)	0.98 (0.83, 1.15)	1.05 (0.89, 1.23)	0.98 (0.82, 1.18)	0.745
Further adjusted for tobacco smoking	1.00	0.98 (0.82, 1.18)	0.96 (0.81, 1.12)	1.01 (0.86, 1.19)	0.93 (0.77, 1.12)	0.675
Further adjusted for alcohol consumption	1.00	0.97 (0.81, 1.17)	0.93 (0.79, 1.10)	0.98 (0.84, 1.16)	0.90 (0.75, 1.09)	0.645
Female breast cancer^d^						
Cases	1552	127	134	125	46	
Cases/PYs (/1000)	0.68	0.80	0.59	0.74	0.65	
Crude model	1.00	1.13 (0.94, 1.36)	1.03 (0.85, 1.25)	1.27 (1.04, 1.55)	0.86 (0.63, 1.17)	0.369
Multivariable-adjusted	1.00	1.02 (0.85, 1.23)	0.96 (0.80, 1.16)	1.16 (0.95, 1.42)	0.79 (0.58, 1.07)	0.267
Further adjusted for tobacco smoking	1.00	1.02 (0.85, 1.23)	0.96 (0.80, 1.16)	1.16 (0.95, 1.42)	0.79 (0.58, 1.07)	0.258
Further adjusted for alcohol consumption	1.00	1.02 (0.85, 1.23)	0.96 (0.79, 1.16)	1.16 (0.95, 1.42)	0.79 (0.58, 1.07)	0.239
Female cervix uteri cancer^e^						
Cases	551	55	130	95	37	
Cases/PYs (/1000)	0.25	0.35	0.59	0.58	0.54	
Crude model	1.00	1.00 (0.75, 1.33)	1.18 (0.94, 1.49)	1.02 (0.79, 1.32)	1.09 (0.76, 1.56)	0.579
Multivariable-adjusted	1.00	0.99 (0.74, 1.32)	1.16 (0.92, 1.46)	1.00 (0.77, 1.29)	1.06 (0.74, 1.51)	0.631
Further adjusted for tobacco smoking	1.00	0.98 (0.74, 1.31)	1.15 (0.92, 1.46)	0.99 (0.77, 1.29)	1.05 (0.73, 1.50)	0.581
Further adjusted for alcohol consumption	1.00	0.98 (0.74, 1.31)	1.15 (0.91, 1.45)	0.99 (0.77, 1.28)	1.05 (0.73, 1.50)	0.566

HR indicates hazard ratio; CI, confidence interval; and PYs, person-years

Multivariable model was adjusted for education (no formal school, primary school, middle school, high school, college, or university or above), occupation (agriculture, industrial, administrative or managerial, professional or technical, sales and service, retired, house wife or husband, self-employed, unemployed, other), marital status (married, widowed, divorced or separated, or never married), household income (RMB/year: < 2500, 2500–4999, 5000–9999, 10,000–19,999, 20,000–34,999, or ≥ 35,000), physical activity (metabolic equivalent of task-hour/day), intakes of red meat, fresh fruits and vegetables (days/week, calculated by assigning participants to the midpoint of their intake category), body mass index (kg/m^2^), waist-hip ratio, family history of cancer (presence or absence), and prevalent diabetes (presence or absence). Multivariable model was further adjusted for tobacco smoking (nonsmokers; current smoking 1–9, 10–19, 20–29, or ≥ 30 cigarettes or equivalent tobacco per day) and alcohol consumption (less than weekly; weekly; daily consuming < 15, 15–29, 30–59, ≥ 60 grams of pure alcohol)

^a^Additionally adjusted for exposure to secondhand smoke (never, occasionally, 1–2 days/week, 3–5 days/week, daily) and its duration (hours/week)

^b^Additionally adjusted for intake of preserved vegetables (days/week, calculated by assigning participants to the midpoint of their intake category)

^c^Additionally adjusted for hepatitis B surface antigen status (positive, negative, or unclear)

^d^Included 289,125 female participants. Additionally adjusted for menopausal status (premenopausal, perimenopausal, or postmenopausal), age at first period (year), ever used oral contraceptives (yes or no), and number of live births

^e^Included 280,243 female participants. Additionally adjusted for menopausal status (premenopausal, perimenopausal, or postmenopausal)

^f^Restricted to daily tea consumers and calculated by assigning the median of tea leaves added to three categories

Similar associations between daily tea consumption and risk of all cancers, lung cancer, and stomach cancer were observed in male participants (Appendix Table 2). Among female participants, however, we did not find association between tea consumption and any cancer risk (Appendix Table 3). In the sensitivity analyses, we excluded participants whose outcome occurred during the first 3 years of follow-up. We additionally adjusted for history of peptic ulcer in analyses of stomach cancer, and history of chronic hepatitis or cirrhosis and gallstone or gallbladder disease in analyses of liver cancer. The risk estimates did not change materially (Appendix Table 4).

### Tea consumption and cancer risk by tobacco smoking and excessive alcohol consumption

We also investigated the association between tea consumption and cancer risk by combined categories of tobacco smoking and excessive alcohol consumption. It was not conducted for female breast cancer and cervix uteri cancer because most female participants in this study neither smoked tobacco nor consumed alcohol excessively. Among those who neither smoked nor consumed alcohol excessively, tea consumption was not associated with all cancers (daily consumers who added tea leaves > 4.0 g/day vs. less-than-weekly consumers: HR, 1.03; CI, 0.93–1.13), lung cancer (HR, 1.08; CI, 0.84–1.40), colorectal cancer (HR, 1.08; CI, 0.81–1.45) and liver cancer (HR, 1.08; CI, 0.75–1.55), yet might be associated with a higher risk of stomach cancer (HR, 1.46; CI, 1.07–1.99) (Table 3; Appendix Table 5). Among those who either smoked or consumed alcohol excessively, daily consumers who added tea leaves 2.1–4.0 g/day and > 4.0 g/day had increased risk of all cancers and lung cancer (both *P*_*interaction*_< 0.05).

**Table 3:** HRs (95% CIs) for the association between tea consumption and cancer risk by tobacco smoking and excessive alcohol consumption among 455,981 participants

	Less than weekly		Weekly		Daily						*P*_*interaction*_
					≤ 2 g		2.1–4.0 g		> 4.0 g		
	Cases	HR	Cases	HR (95%CI)	Cases	HR (95%CI)	Cases	HR (95%CI)	Cases	HR (95%CI)	
All cancers											< 0.001
Smoking (−) and excessive alcohol consumption (−)	11,082	1.00	840	1.07 (0.99, 1.15)	1183	0.99 (0.93, 1.06)	975	1.04 (0.97, 1.12)	447	1.03 (0.93, 1.13)	
Smoking (+) or excessive alcohol consumption (+)	3390	1.00	653	1.00 (0.92, 1.09)	1258	1.06 (0.99, 1.14)	1354	1.12 (1.04, 1.20)	1470	1.25 (1.17, 1.34)	
Lung cancer											0.010
Smoking (−) and excessive alcohol consumption (−)	1488	1.00	114	1.06 (0.87, 1.30)	180	0.96 (0.81, 1.14)	138	0.95 (0.78, 1.16)	68	1.08 (0.84, 1.40)	
Smoking (+) or excessive alcohol consumption (+)	790	1.00	134	0.89 (0.74, 1.07)	323	1.10 (0.96, 1.27)	375	1.23 (1.08, 1.42)	436	1.51 (1.33, 1.72)	
Stomach cancer											0.702
Smoking (−) and excessive alcohol consumption (−)	965	1.00	62	1.06 (0.82, 1.39)	67	0.81 (0.63, 1.05)	54	0.86 (0.65, 1.15)	47	1.46 (1.07, 1.99)	
Smoking (+) or excessive alcohol consumption (+)	491	1.00	111	1.10 (0.88, 1.36)	172	0.90 (0.75, 1.09)	167	0.87 (0.72, 1.05)	230	1.15 (0.97, 1.37)	
Colorectal cancer											0.774
Smoking (−) and excessive alcohol consumption (−)	1141	1.00	95	1.17 (0.94, 1.45)	144	1.16 (0.96, 1.40)	100	1.06 (0.85, 1.33)	52	1.08 (0.81, 1.45)	
Smoking (+) or excessive alcohol consumption (+)	290	1.00	74	1.17 (0.90, 1.53)	110	1.00 (0.78, 1.27)	116	1.01 (0.80, 1.28)	145	1.22 (0.98, 1.52)	
Liver cancer											0.895
Smoking (−) and excessive alcohol consumption (−)	758	1.00	52	0.93 (0.69, 1.24)	82	0.94 (0.73, 1.21)	73	1.00 (0.77, 1.31)	33	1.08 (0.75, 1.55)	
Smoking (+) or excessive alcohol consumption (+)	385	1.00	86	1.05 (0.82, 1.34)	134	1.02 (0.82, 1.27)	150	1.06 (0.86, 1.32)	121	0.91 (0.73, 1.14)	

HR indicates hazard ratio; CI, confidence interval

Multivariable model was adjusted for the same set of covariates as in the Table 2

Case/person-years information was listed in the Appendix Table 5

Smoking (−): nonsmoking; (+): current smoking

Excessive alcohol consumption (−): less than daily or < 15 g/d of pure alcohol consumption; (+): ≥ 15 g/d of pure alcohol consumption

### Tobacco smoking or alcohol consumption and cancer risk by tea consumption

**Fig. 1:**
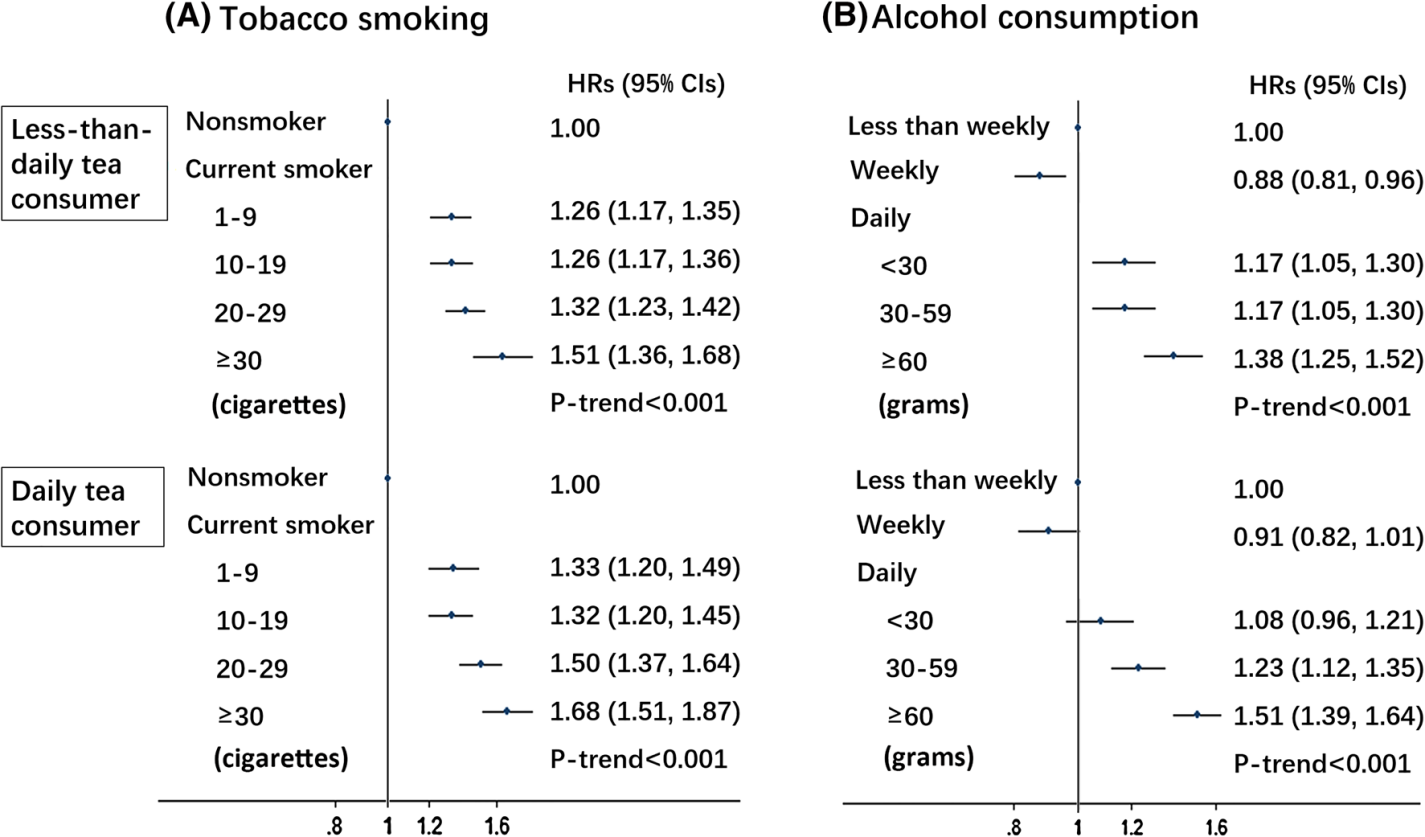
HRs (95% CIs) for all cancer risk in relation to tobacco smoking (**a**) and alcohol consumption (**b**) by tea consumption frequency among 455,981 participants. Multivariable model was adjusted for the same set of covariates for all cancer analysis as in the Table 2. The amount of alcohol and tobacco were given in grams and cigarette or equivalent tobacco, respectively

We further examined the association of all cancers risk with tobacco smoking or alcohol consumption across strata of tea consumption frequency. In both less-than-daily tea consumers and daily tea consumers, all cancers risk increased with the amount of tobacco smoked (Fig. 1**a**) or alcohol consumed (Fig. 1**b**) (all *P*_*trend*_< 0.001).

## Discussion

### Principal findings

In this large prospective Chinese cohort, among daily tea consumers who neither smoked nor consumed alcohol excessively, tea consumption was not associated with risks of all cancers, lung cancer, colorectal cancer, liver cancer, female breast cancer and cervix uteri cancer. Stronger tea consumption as > 4.0 g tea leaves added per day might be associated with an increased risk of stomach cancer. Besides, smoking tobacco and drinking alcohol excessively were consistently associated with elevated cancer risk, no matter the status of tea consumption.

### Comparison with other studies and potential mechanisms

After restricting analyses to both non-smokers and non-excessive drinkers and effectively removing confounding potentials by smoking and excessive alcohol drinking, we did not observe association between tea consumption and lung cancer, colorectal cancer, liver cancer, and female cervix uteri cancer. Our findings are consistent with recent meta-analyses that only included prospective observational studies [18, 19]. The null finding regarding female breast cancer is also in line with the conclusion from the latest meta-analyses [20]. A previous meta-analysis that included large proportion of case–control studies and indicated the protective or harmful effects of tea was rather vulnerable to recall bias and reverse causality [21].

The only Chinese cohorts on this topic were Shanghai Women’s Health Study (SWHS), Shanghai Men’s Health Study (SMHS) and Chinese Prospective Smoking Study (CPSS). SWHS, conducted in 69,310 non-smoking and non-alcohol drinking women, observed reduced risk of digestive system cancers among tea consumers who added tea leaves ≥ 5 g/day (HR, 0.79; CI, 0.63–0.99) after a mean follow-up of 11 years [4]. SMHS of 61,491 men particularly stratified the analysis of green tea consumption and colorectal cancer risk by smoking status. Non-smoking tea consumers who added tea leaves ≥ 8 g/day were shown to have a lower risk of colorectal cancer (HR, 0.44; CI, 0.22–0.88) than never consumers during 5 years of follow-up [22]. However, only ten colorectal cancer cases in this subgroup added to the need for cautious interpretation. A study based on these two cohorts with extensive follow-up did not find any association between green tea consumption and cancer mortality in SWHS, SMHS, and two cohorts combined [23]. CPSS followed 164,681 men for 11 years and found decrease in risk of cancer mortality in non-smokers who consumed tea leaves ≤ 5 g/day compared with non-consumers. However, this trend did not appear in non-smokers who consumed higher amount of tea [24].

The effect of tea consumption on stomach cancer is still in debate. A cohort study of 3158 Japanese with a mean follow-up of 14 years has shown a higher risk of stomach cancer among female weekly consumers of black tea than less-than-weekly consumers (HR, 3.8; CI, 1.1–13.6) [8]. Yet most recent meta-analyses did not find association between tea consumption and stomach cancer among cohort studies [25, 26]. A few case–control studies suggested that the temperature might be influential and drinking hot tea was associated with increased risk of stomach cancer [27, 28]. Another potential explanation is the caffeine in tea stimulating gastric acid secretion. The finding of increased risk of stomach cancer among coffee consumers but not in de-caffeinated coffee consumers might partly corroborate this assumption [5]. In the current study, we only observed increased stomach cancer risk in the highest tea consumption group among non-smokers and non-excessive alcohol consumers. As cases in that specific group were limited, the significant result could also be due solely to the play of chance. A possible reason of association not observed in tea consumers who smoked or consumed alcohol excessively is that they already gained a high baseline risk due to smoking and alcohol use, so even if tea consumption was harmful, the additional risk would be obscured.

Tobacco smoking and excessive alcohol consumption, as established risk factors for cancer, were closely correlated with tea consumption in our study population, especially among male participants. The substantial attenuation of HRs appeared when additionally adjusting for smoking in multivariable models, indicating that the heightened risks of all cancers and lung cancer associated with daily tea consumption observed in all participants and men were more likely the results of confounding from smoking. The possibility of residual confounding by smoking intensity was eliminated after restricting the analyses to non-smokers. On the other hand, we did not find any effect of tea consumption on antagonizing tobacco- or alcohol-induced cancer in the present population. Therefore, the public should be informed that smoking cessation and limiting alcohol drinking are much more reliable approaches to preventing cancer than relying on some particular foods or beverages.

### Strengths and limitations

To our knowledge, this is by far the largest prospective study assessing the association of tea consumption with overall and site-specific cancer incidence. The large sample size enabled us to minimize residual confounding by restricting analyses to non-smokers and non-excessive alcohol consumers upon comprehensive adjustment of other potential confounders. Inclusion of a geographically spread population living in urban and rural areas made the current study represent a wider range of Chinese population than previous ones. We measured tea consumption with the grams of tea leaves added, which may reflect the amount of bioactive components in tea and be a better measurement of tea consumption than cups of tea. We also excluded participants who had reduced their use of tea, tobacco, and alcohol, and even excluded incident cases in the first 3 years of follow-up to avoid potential reverse causality.

Several limitations of our study should also be considered. First, tea consumption information was self-reported and collected once at baseline, hardly considering changes in consumption habits, raising possibility of misclassification. However, exposure misclassification were more likely to be non-differential on subsequent disease status. Second, we cannot rule out residual confounding by other unmeasured or unknown factors, coffee consumption, for example. Nevertheless, less than 2% of our participants consumed coffee weekly, based on a re-survey involving about 5% of randomly chosen surviving CKB participants. Third, brewing methods and tea types might also influence the amount of tea polyphenols [29], which could not be fully captured by only using tea leaved added. We are unable to confirm whether the results are consistent across brewing methods or tea types due to brewing methods information unavailable and most participants drinking green tea.

## Conclusion

Results from this large prospective study of Chinese population did not indicate association between tea consumption and cancer risk among non-smokers and non-excessive alcohol users except for stomach cancer. Lifestyle modifications like smoking cessation and limited alcohol consumption may offer more benefit on cancer prevention than tea consumption. Future studies on the relationship between tea consumption and stomach cancer are warranted.

## Electronic supplementary material

Below is the link to the electronic supplementary material.

Supplementary material 1 (DOCX 64 kb)
