# Supplementary material for: Association between tea consumption and risk of cancer: a prospective cohort study of 0.5 million Chinese adults
Source: Eur J Epidemiol. 2019 May 31;34(8):753–63. doi: 10.1007/s10654-019-00530-5 (PMC6602977; doi:10.1007/s10654-019-00530-5)
Supplement: Supplementary file 1 — Supplementary material 1 (DOCX 64 kb) [file 10654_2019_530_MOESM1_ESM.docx]

**Supplementary Appendix**

**Table of contents**

[**Members of the China Kadoorie Biobank collaborative group** 1](#_Toc8331112)

[**Appendix table 1.** Other baseline characteristics of 455,981 participants according to tea consumption. 3](#_Toc8331113)

[**Appendix table 2.** HRs (95%CIs) for the association between tea consumption and cancer risk among 164,480 male participants. 5](#_Toc8331114)

[**Appendix table 3.** HRs (95%CIs) for the association between tea consumption and cancer risk among 291,501 female participants. 9](#_Toc8331115)

[**Appendix table 4.** HRs (95%CIs) for the sensitivity analyses among 455,981 participants. 13](#_Toc8331116)

[**STROBE Statement**—checklist of items that should be included in reports of observational studies 20](#_Toc8331117)

**Members of the China Kadoorie Biobank collaborative group**

**International Steering Committee:** Junshi Chen, Zhengming Chen (PI), Robert Clarke, Rory Collins, Yu Guo, Liming Li (PI), Jun Lv, Richard Peto, Robin Walters. **International Co-ordinating Centre, Oxford:** Daniel Avery, Ruth Boxall, Derrick Bennett, Yumei Chang, Yiping Chen, Zhengming Chen, Robert Clarke, Huaidong Du, Simon Gilbert, Alex Hacker, Mike Hill, Michael Holmes, Andri Iona, Christiana Kartsonaki, Rene Kerosi, Ling Kong, Om Kurmi, Garry Lancaster, Sarah Lewington, Kuang Lin, John McDonnell, Iona Millwood, Qunhua Nie, Jayakrishnan Radhakrishnan, Paul Ryder, Sam Sansome, Dan Schmidt, Paul Sherliker, Rajani Sohoni, Becky Stevens, Iain Turnbull, Robin Walters, Jenny Wang, Lin Wang, Neil Wright, Ling Yang, Xiaoming Yang. **National Co-ordinating Centre, Beijing:** Zheng Bian, Yu Guo, Xiao Han, Can Hou, Jun Lv, Pei Pei, Chao Liu, Yunlong Tan, Canqing Yu. **10 Regional Co-ordinating Centres: Qingdao CDC:** Zengchang Pang, Ruqin Gao, Shanpeng Li, Shaojie Wang, Yongmei Liu, Ranran Du, Yajing Zang, Liang Cheng, Xiaocao Tian, Hua Zhang, Yaoming Zhai, Feng Ning, Xiaohui Sun, Feifei Li. **Licang CDC:** Silu Lv, Junzheng Wang, Wei Hou. **Heilongjiang Provincial CDC:** Mingyuan Zeng, Ge Jiang, Xue Zhou. **Nangang CDC:** Liqiu Yang, Hui He, Bo Yu, Yanjie Li, Qinai Xu,Quan Kang, Ziyan Guo. **Hainan Provincial CDC:** Dan Wang, Ximin Hu, Jinyan Chen, Yan Fu, Zhenwang Fu, Xiaohuan Wang. **Meilan CDC:** Min Weng, Zhendong Guo, Shukuan Wu,Yilei Li, Huimei Li, Zhifang Fu. **Jiangsu Provincial CDC:** Ming Wu, Yonglin Zhou, Jinyi Zhou, Ran Tao, Jie Yang, Jian Su. **Suzhou CDC:** Fang liu, Jun Zhang, Yihe Hu, Yan Lu, , Liangcai Ma, Aiyu Tang, Shuo Zhang, Jianrong Jin, Jingchao Liu. **Guangxi Provincial CDC:** Zhenzhu Tang, Naying Chen, Ying Huang. **Liuzhou CDC:** Mingqiang Li, Jinhuai Meng, Rong Pan, Qilian Jiang, Jian Lan,Yun Liu, Liuping Wei, Liyuan Zhou, Ningyu Chen Ping Wang, Fanwen Meng, Yulu Qin,, Sisi Wang. **Sichuan Provincial CDC:** Xianping Wu, Ningmei Zhang, Xiaofang Chen,Weiwei Zhou. **Pengzhou CDC:** Guojin Luo, Jianguo Li, Xiaofang Chen, Xunfu Zhong, Jiaqiu Liu, Qiang Sun. **Gansu Provincial CDC:** Pengfei Ge, Xiaolan Ren, Caixia Dong. **Maiji CDC:** Hui Zhang, Enke Mao, Xiaoping Wang, Tao Wang, Xi zhang. **Henan Provincial CDC:** Ding Zhang, Gang Zhou, Shixian Feng, Liang Chang, Lei Fan. **Huixian CDC:** Yulian Gao, Tianyou He, Huarong Sun, Pan He, Chen Hu, Xukui Zhang, Huifang Wu, Pan He. **Zhejiang Provincial CDC:** Min Yu, Ruying Hu, Hao Wang. Tongxiang CDC: Yijian Qian, Chunmei Wang, Kaixu Xie, Lingli Chen, Yidan Zhang, Dongxia Pan, Qijun Gu. **Hunan Provincial CDC:** Yuelong Huang, Biyun Chen, Li Yin, Huilin Liu, Zhongxi Fu, Qiaohua Xu. **Liuyang CDC:** Xin Xu, Hao Zhang, Huajun Long, Xianzhi Li, Libo Zhang, Zhe Qiu.

**Appendix table 1. Other baseline characteristics of 455,981 participants according to tea consumption.**

|  | Less than weekly | Weekly | Daily |  |  | *P_trend_* |
| --- | --- | --- | --- | --- | --- | --- |
|  |  |  | ≤2.0g | 2.1-4.0g | ＞4.0g |  |
| Middle school and above, % | 47.6 | 52.9 | 51.7 | 53.3 | 52.8 | <0.001 |
| Non-agricultural occupation, % | 56.7 | 59.8 | 59.0 | 59.5 | 61.7 | <0.001 |
| Household income day ≥20,000 Renminbi per year, % | 41.0 | 46.0 | 43.3 | 45.1 | 46.1 | <0.001 |
| Married, % | 90.2 | 90.8 | 91.3 | 91.6 | 91.9 | <0.001 |
| Physical activity, MET-hour/day | 21.8 | 21.3 | 20.5 | 20.4 | 20.2 | <0.001 |
| Average weekly intake, days^a^ |  |  |  |  |  |  |
| Red meat | 3.6 | 3.8 | 3.9 | 3.8 | 4.0 | <0.001 |
| Fresh fruits | 2.5 | 2.8 | 2.9 | 2.8 | 2.8 | <0.001 |
| Fresh vegetables | 6.8 | 6.8 | 6.9 | 6.9 | 6.9 | <0.001 |
| Preserved vegetables | 2.1 | 2.2 | 2.2 | 2.2 | 2.3 | <0.001 |
| Body mass index, kg/m^2^ | 23.5 | 23.8 | 23.8 | 23.9 | 24.0 | <0.001 |
| Waist hip ratio | 0.88 | 0.88 | 0.88 | 0.88 | 0.88 | <0.001 |
| Secondhand smoke exposure, % | 59.9 | 70.5 | 69.6 | 69.5 | 72.7 | <0.001 |
| Average duration of secondhand smoke exposure, hours | 10.0 | 10.2 | 12.0 | 11.8 | 13.6 | <0.001 |
| Family history of cancer, % | 16.2 | 17.4 | 17.0 | 17.2 | 19.4 | <0.001 |
| History of diabetes, % | 5.4 | 5.7 | 5.8 | 5.8 | 6.3 | <0.001 |
| History of peptic ulcer, % | 3.8 | 3.3 | 3.2 | 2.9 | 3.3 | <0.001 |
| History of cirrhosis/chronic hepatitis, % | 1.2 | 1.0 | 1.0 | 0.8 | 1.1 | <0.001 |
| History of gallstone/gallbladder disease, % | 6.0 | 6.2 | 5.9 | 5.7 | 5.9 | 0.175 |
| HBsAg positive, % | 3.1 | 3.0 | 2.9 | 2.8 | 2.8 | <0.001 |
| Postmenopausal in women, % | 51.9 | 51.3 | 51.3 | 50.9 | 50.7 | <0.001 |
| Average age at first period, year | 15.5 | 15.3 | 15.4 | 15.3 | 15.4 | <0.001 |
| Ever used oral contraceptives, % | 9.6 | 10.3 | 10.7 | 10.6 | 10.9 | <0.001 |
| Average number of live births | 2.2 | 2.2 | 2.2 | 2.2 | 2.2 | <0.001 |

MET indicates metabolic equivalent of task; HBsAg, hepatitis B surface antigen. The results are presented as adjusted means or percentages, with adjustment for age, sex, and study area, as appropriate.

^a^ Average weekly intake of red meat, fresh fruits and vegetables, and preserved vegetables were calculated by assigning participants to the midpoint of their intake category (daily, 4-6 days/week, 1-3 days/week, monthly, or rarely or never).

**Appendix table 2. HRs (95%CIs) for the association between tea consumption and cancer risk among 164,480 male participants.**

|  | Less than weekly | Weekly | Daily | | | *P_trend_* ^d^ |
| --- | --- | --- | --- | --- | --- | --- |
|  |  |  | ≤2.0g | 2.1-4.0g | ＞4.0g |  |
| **All cancers** |  |  |  |  |  |  |
| Cases | 4,349 | 784 | 1,381 | 1,519 | 1,576 |  |
| Cases/PYs (/1,000) | 5.72 | 4.74 | 6.55 | 6.33 | 7.10 |  |
| Multivariable-adjusted | 1.00 | 1.04 (0.96, 1.12) | 1.08 (1.01, 1.16) | 1.16 (1.09, 1.24) | 1.33 (1.25, 1.42) | <0.001 |
| Further adjusted for tobacco smoking | 1.00 | 1.00 (0.92, 1.08) | 1.02 (0.96, 1.09) | 1.08 (1.01, 1.15) | 1.19 (1.11, 1.27) | <0.001 |
| Further adjusted for alcohol consumption | 1.00 | 0.99 (0.91, 1.07) | 1.00 (0.93, 1.07) | 1.05 (0.98, 1.12) | 1.15 (1.08, 1.23) | <0.001 |
| **Lung cancer**^a^ |  |  |  |  |  |  |
| Cases | 848 | 152 | 343 | 391 | 451 |  |
| Cases/PYs (/1,000) | 1.11 | 0.91 | 1.61 | 1.61 | 2.01 |  |
| Multivariable-adjusted | 1.00 | 0.95 (0.79, 1.13) | 1.18 (1.03, 1.35) | 1.30 (1.14, 1.48) | 1.68 (1.49, 1.91) | <0.001 |
| Further adjusted for tobacco smoking | 1.00 | 0.88 (0.73, 1.05) | 1.06 (0.93, 1.22) | 1.12 (0.98, 1.27) | 1.34 (1.18, 1.53) | 0.001 |
| Further adjusted for alcohol consumption | 1.00 | 0.88 (0.73, 1.05) | 1.06 (0.92, 1.21) | 1.11 (0.97, 1.26) | 1.33 (1.17, 1.51) | 0.002 |
| **Stomach cancer**^b^ |  |  |  |  |  |  |
| Cases | 672 | 134 | 190 | 185 | 252 |  |
| Cases/PYs (/1,000) | 0.88 | 0.80 | 0.89 | 0.76 | 1.12 |  |
| Multivariable-adjusted | 1.00 | 1.18 (0.97, 1.44) | 0.96 (0.80, 1.14) | 0.92 (0.78, 1.10) | 1.29 (1.10, 1.52) | 0.001 |
| Further adjusted for tobacco smoking | 1.00 | 1.14 (0.94, 1.39) | 0.91 (0.76, 1.09) | 0.87 (0.73, 1.04) | 1.19 (1.01, 1.40) | 0.002 |
| Further adjusted for alcohol consumption | 1.00 | 1.14 (0.93, 1.38) | 0.90 (0.75, 1.07) | 0.86 (0.72, 1.02) | 1.17 (1.00, 1.38) | 0.002 |
| **Colorectal cancer** |  |  |  |  |  |  |
| Cases | 422 | 94 | 133 | 136 | 159 |  |
| Cases/PYs (/1,000) | 0.55 | 0.56 | 0.62 | 0.56 | 0.71 |  |
| Multivariable-adjusted | 1.00 | 1.14 (0.90, 1.43) | 1.01 (0.82, 1.25) | 0.98 (0.80, 1.21) | 1.20 (0.99, 1.46) | 0.056 |
| Further adjusted for tobacco smoking | 1.00 | 1.11 (0.88, 1.40) | 0.98 (0.79, 1.21) | 0.95 (0.77, 1.17) | 1.15 (0.94, 1.40) | 0.092 |
| Further adjusted for alcohol consumption | 1.00 | 1.09 (0.87, 1.38) | 0.95 (0.77, 1.18) | 0.92 (0.75, 1.13) | 1.11 (0.91, 1.36) | 0.110 |
| **Liver cancer**^c^ |  |  |  |  |  |  |
| Cases | 540 | 101 | 147 | 184 | 134 |  |
| Cases/PYs (/1,000) | 0.70 | 0.61 | 0.69 | 0.76 | 0.60 |  |
| Multivariable-adjusted | 1.00 | 1.00 (0.80, 1.25) | 0.97 (0.79, 1.18) | 1.15 (0.95, 1.38) | 0.96 (0.78, 1.18) | 0.497 |
| Further adjusted for tobacco smoking | 1.00 | 0.97 (0.78, 1.21) | 0.93 (0.76, 1.14) | 1.10 (0.91, 1.32) | 0.90 (0.73, 1.10) | 0.452 |
| Further adjusted for alcohol consumption | 1.00 | 0.96 (0.77, 1.21) | 0.91 (0.74, 1.12) | 1.06 (0.88, 1.28) | 0.87 (0.71, 1.07) | 0.437 |

HR indicates hazard ratio; CI, confidence interval; and PYs, person-years.

Multivariable model was adjusted for education (no formal school, primary school, middle school, high school, college, or university or above), occupation (agriculture and related workers, factory worker, administrator or manager, professional or technical, sales and service workers, retired, house wife or husband, self-employed, unemployed, other), marital status (married, widowed, divorced/separated, or never married), household income (Chinese Renminbi/year: <2,500, 2,500-4,999, 5,000-9,999, 10,000-19,999, 20,000-34,999, or ≥35,000), physical activity (metabolic equivalent of task-hour/day), intakes of red meat, fresh fruits and vegetables (days/week, calculated by assigning participants to the midpoint of their intake category), body mass index (kg/m^2^),waist-hip ratio, family history of cancer (presence of absence), and history of diabetes (presence or absence). Multivariable model was further adjusted for tobacco smoking (nonsmokers, current smokes 1-9, 10-19, 20-29, or ≥30 cigarettes or equivalents per day) and alcohol consumption (less than weekly, weekly, daily <15, 15-29, 30-59, ≥60 grams of pure alcohol).

^a^ Additionally adjusted for secondhand smoke exposure frequency (never, occasionally, 1-2, 3-5 days per week, daily) and duration (hours).

^b^ Additionally adjusted for intake of preserved vegetables (days/week, calculated by assigning participants to the midpoint of their intake category).

^c^ Additionally adjusted for hepatitis B test result (positive, negative, unclear, missing).

^d^ Restricted to daily tea consumers and calculated by assigning the median of tea leaves added to three categories.

**Appendix table 3. HRs (95%CIs) for the association between tea consumption and cancer risk among 291,501 female participants.**

|  | Less than weekly | Weekly | Daily | | | *P_trend_* ^d^ |
| --- | --- | --- | --- | --- | --- | --- |
|  |  |  | ≤2.0g | 2.1-4.0g | ＞4.0g |  |
| **All cancers** |  |  |  |  |  |  |
| Cases | 10,123 | 709 | 1,060 | 810 | 341 |  |
| Cases/PYs (/1,000) | 4.46 | 4.45 | 4.65 | 4.83 | 4.80 |  |
| Multivariable-adjusted | 1.00 | 1.08 (1.00, 1.17) | 1.00 (0.93, 1.08) | 1.05 (0.97, 1.14) | 1.00 (0.90, 1.12) | 0.867 |
| Further adjusted for tobacco smoking | 1.00 | 1.07 (0.99, 1.16) | 1.00 (0.93, 1.07) | 1.04 (0.96, 1.13) | 0.99 (0.89, 1.11) | 0.977 |
| Further adjusted for alcohol consumption | 1.00 | 1.07 (0.99, 1.16) | 1.00 (0.93, 1.07) | 1.04 (0.96, 1.13) | 0.99 (0.89, 1.11) | 0.945 |
| **Lung cancer**^a^ |  |  |  |  |  |  |
| Cases | 1,430 | 96 | 160 | 122 | 53 |  |
| Cases/PYs (/1,000) | 0.62 | 0.59 | 0.69 | 0.72 | 0.74 |  |
| Multivariable-adjusted | 1.00 | 1.11 (0.90, 1.38) | 1.00 (0.83, 1.21) | 1.08 (0.88, 1.34) | 1.15 (0.86, 1.53) | 0.342 |
| Further adjusted for tobacco smoking | 1.00 | 1.08 (0.87, 1.33) | 0.97 (0.81, 1.17) | 1.03 (0.83, 1.28) | 1.08 (0.81, 1.44) | 0.465 |
| Further adjusted for alcohol consumption | 1.00 | 1.09 (0.89, 1.35) | 0.98 (0.82, 1.18) | 1.04 (0.84, 1.29) | 1.09 (0.82, 1.46) | 0.450 |
| **Stomach cancer**^b^ |  |  |  |  |  |  |
| Cases | 784 | 39 | 49 | 36 | 25 |  |
| Cases/PYs (/1,000) | 0.34 | 0.24 | 0.21 | 0.21 | 0.35 |  |
| Multivariable-adjusted | 1.00 | 0.99 (0.71, 1.37) | 0.81 (0.60, 1.09) | 0.95 (0.67, 1.36) | 1.46 (0.96, 2.22) | 0.003 |
| Further adjusted for tobacco smoking | 1.00 | 0.98 (0.71, 1.36) | 0.80 (0.59, 1.09) | 0.95 (0.67, 1.35) | 1.44 (0.95, 2.20) | 0.003 |
| Further adjusted for alcohol consumption | 1.00 | 0.98 (0.70, 1.36) | 0.80 (0.59, 1.09) | 0.95 (0.66, 1.35) | 1.44 (0.95, 2.20) | 0.003 |
| **Colorectal cancer** |  |  |  |  |  |  |
| Cases | 1,009 | 75 | 121 | 80 | 38 |  |
| Cases/PYs (/1,000) | 0.44 | 0.46 | 0.53 | 0.47 | 0.53 |  |
| Multivariable-adjusted | 1.00 | 1.19 (0.94, 1.51) | 1.15 (0.93, 1.42) | 1.11 (0.86, 1.44) | 1.11 (0.79, 1.56) | 0.979 |
| Further adjusted for tobacco smoking | 1.00 | 1.19 (0.94, 1.52) | 1.15 (0.93, 1.42) | 1.12 (0.87, 1.44) | 1.12 (0.79, 1.57) | 0.983 |
| Further adjusted for alcohol consumption | 1.00 | 1.20 (0.94, 1.53) | 1.16 (0.94, 1.43) | 1.12 (0.87, 1.45) | 1.12 (0.79, 1.57) | 0.899 |
| **Liver cancer**^c^ |  |  |  |  |  |  |
| Cases | 603 | 37 | 69 | 39 | 20 |  |
| Cases/PYs (/1,000) | 0.26 | 0.23 | 0.30 | 0.23 | 0.28 |  |
| Multivariable-adjusted | 1.00 | 1.02 (0.72, 1.43) | 1.05 (0.79, 1.40) | 0.78 (0.54, 1.12) | 1.15 (0.72, 1.84) | 0.803 |
| Further adjusted for tobacco smoking | 1.00 | 1.01 (0.72, 1.43) | 1.04 (0.78, 1.39) | 0.78 (0.54, 1.12) | 1.14 (0.71, 1.83) | 0.890 |
| Further adjusted for alcohol consumption | 1.00 | 1.01 (0.72, 1.43) | 1.05 (0.79, 1.40) | 0.78 (0.54, 1.12) | 1.14 (0.71, 1.83) | 0.927 |

HR indicates hazard ratio; CI, confidence interval; and PYs, person-years. Analyses of female breast cancer and cervix uteri cancer were shown in Table 2.

Multivariable model was adjusted for education (no formal school, primary school, middle school, high school, college, or university or above), occupation (agriculture and related workers, factory worker, administrator or manager, professional or technical, sales and service workers, retired, house wife or husband, self-employed, unemployed, other), marital status (married, widowed, divorced/separated, or never married), household income (Chinese Renminbi/year: <2,500, 2,500-4,999, 5,000-9,999, 10,000-19,999, 20,000-34,999, or ≥35,000), physical activity (metabolic equivalent of task-hour/day), intakes of red meat, fresh fruits and vegetables (days/week, calculated by assigning participants to the midpoint of their intake category), body mass index (kg/m^2^),waist hip ratio, family history of cancer (presence of absence), history of diabetes (presence or absence) and menopausal status (premenopausal, perimenopausal, or postmenopausal). Multivariable model was further adjusted for tobacco smoking (nonsmokers, current smokes 1-9, 10-19, 20-29, or ≥30 cigarettes or equivalents per day) and alcohol consumption (less than weekly, weekly, daily <15, 15-29, 30-59, ≥60 grams of pure alcohol).

^a^ Additionally adjusted for secondhand smoke exposure frequency (never, occasionally, 1-2, 3-5 days per week, daily) and duration (hours).

^b^ Additionally adjusted for intake of preserved vegetables (days/week, calculated by assigning participants to the midpoint of their intake category).

^c^ Additionally adjusted for hepatitis B test result (positive, negative, unclear, missing).

^d^ Restricted to daily tea consumers and calculated by assigning the median of tea leaves added to three categories.

**Appendix table 4. HRs (95%CIs) for the sensitivity analyses among 455,981 participants.**

|  | Less than weekly | Weekly | Daily | | |
| --- | --- | --- | --- | --- | --- |
|  |  |  | ≤2.0g | 2.1-4.0g | ＞4.0g |
| **All cancers** |  |  |  |  |  |
| Excluding incident cases in first three years | 1.00 | 1.06 (1.00, 1.13) | 1.03 (0.97, 1.08) | 1.07 (1.01, 1.13) | 1.14 (1.07, 1.21) |
| **Lung cancer**^a^ |  |  |  |  |  |
| Excluding incident cases in first three years | 1.00 | 1.02 (0.85, 1.22) | 1.00 (0.86, 1.17) | 1.07 (0.92, 1.24) | 1.22 (1.04, 1.43) |
| **Stomach cancer**^b^ |  |  |  |  |  |
| Excluding incident cases in first three years | 1.00 | 1.19 (0.99, 1.44) | 0.87 (0.73, 1.04) | 0.88 (0.73, 1.05) | 1.25 (1.05, 1.48) |
| Further adjusting for history of peptic ulcer | 1.00 | 1.08 (0.91, 1.27) | 0.87 (0.75, 1.01) | 0.85 (0.73, 0.99) | 1.16 (1.00, 1.35) |
| **Colorectal cancer** |  |  |  |  |  |
| Excluding incident cases in first three years | 1.00 | 1.13 (0.94, 1.36) | 0.98 (0.83, 1.15) | 0.94 (0.79, 1.12) | 1.13 (0.94, 1.35) |
| **Liver cancer**^c^ |  |  |  |  |  |
| Excluding incident cases in first three years | 1.00 | 1.00 (0.81, 1.23) | 0.88 (0.73, 1.06) | 0.95 (0.79, 1.14) | 0.82 (0.66, 1.02) |
| Further adjusting for history of chronic hepatitis or cirrhosis and gallstone or gallbladder disease | 1.00 | 0.95 (0.79, 1.14) | 0.93 (0.79, 1.09) | 0.97 (0.83, 1.14) | 0.87 (0.73, 1.05) |
| **Female breast cancer**^d^ |  |  |  |  |  |
| Excluding incident cases in first three years | 1.00 | 0.99 (0.80, 1.22) | 1.07 (0.87, 1.31) | 1.26 (1.01, 1.56) | 0.68 (0.47, 0.99) |
| **Female cervix uteri cancer**^e^ |  |  |  |  |  |
| Excluding incident cases in first three years | 1.00 | 1.14 (0.83, 1.57) | 1.28 (0.99, 1.67) | 1.16 (0.87, 1.54) | 1.23 (0.83, 1.83) |

HR indicates hazard ratio; and CI, confidence interval.

Based on multivariable model which adjusted for education (no formal school, primary school, middle school, high school, college, or university or above), occupation (agriculture and related workers, factory worker, administrator or manager, professional or technical, sales and service workers, retired, house wife or husband, self-employed, unemployed, other), marital status (married, widowed, divorced/separated, or never married), household income (Chinese Renminbi/year: <2,500, 2,500-4,999, 5,000-9,999, 10,000-19,999, 20,000-34,999, or ≥35,000), physical activity (metabolic equivalent of task-hour/day), intakes of red meat, fresh fruits and vegetables (days/week, calculated by assigning participants to the midpoint of their intake category), body mass index (kg/m^2^),waist-hip ratio, family history of cancer (presence of absence), and history of diabetes (presence or absence). Multivariable model was further adjusted for tobacco smoking (nonsmokers, current smokes 1-9, 10-19, 20-29, or ≥30 cigarettes or equivalents per day) and alcohol consumption (less than weekly, weekly, daily <15, 15-29, 30-59, ≥60 grams of pure alcohol).

^a^ Additionally adjusted for secondhand smoke exposure frequency (never, occasionally, 1-2, 3-5 days per week, daily) and duration (hours).

^b^ Additionally adjusted for intake of preserved vegetables (days/week, calculated by assigning participants to the midpoint of their intake category).

^c^ Additionally adjusted for hepatitis B test result (positive, negative, unclear, missing).

^d^ Included 289,125 female participants. Additionally adjusted for menopausal status (premenopausal, perimenopausal, or postmenopausal), age at first period (year), ever used oral contraceptives (yes or no), and number of live births.

^e^ Included 280,243 female participants. Additionally adjusted for menopausal status (premenopausal, perimenopausal, or postmenopausal).

**Appendix table 5. Cases and cases/person-years for the association between tea consumption and cancer risk by tobacco smoking and excessive alcohol consumption among 455,981 participants.**

|  | Less than weekly | |  |  | Weekly | | |  | | Daily | | | | | | | | | |
| --- | --- | --- | --- | --- | --- | --- | --- | --- | --- | --- | --- | --- | --- | --- | --- | --- | --- | --- | --- |
|  |  |  |  |  |  |  |  |  | | ≤2g | | |  | | 2.1-4.0g | |  | ＞4.0g | |
|  | Cases | Cases/PYs (/1,000) |  |  | Cases | Cases/PYs (/1,000) |  | | Cases | | Cases/PYs (/1,000) |  | | Cases | | Cases/PYs (/1,000) |  | Cases | Cases/PYs (/1,000) |
| **All cancers** |  |  |  |  |  |  |  | |  | |  |  | |  | |  |  |  |  |
| Smoking (-) and  excessive alcohol consumption (-) | 11,082 | 4.45 |  |  | 840 | 4.26 |  | | 1,183 | | 4.54 |  | | 975 | | 4.73 |  | 447 | 4.77 |
| Smoking (+) or  excessive alcohol consumption (+) | 3,390 | 36.93 |  |  | 653 | 21.64 |  | | 1,258 | | 23.65 |  | | 1,354 | | 23.92 |  | 1,470 | 23.40 |
| **Lung cancer** |  |  |  |  |  |  |  | |  | |  |  | |  | |  |  |  |  |
| Smoking (-) and  excessive alcohol consumption (-) | 1,488 | 0.59 |  |  | 114 | 0.57 |  | | 180 | | 0.68 |  | | 138 | | 0.66 |  | 68 | 0.72 |
| Smoking (+) or  excessive alcohol consumption (+) | 790 | 8.51 |  |  | 134 | 4.40 |  | | 323 | | 6.00 |  | | 375 | | 6.55 |  | 436 | 6.83 |
| **Stomach cancer** |  |  |  |  |  |  |  | |  | |  |  | |  | |  |  |  |  |
| Smoking (-) and  excessive alcohol consumption (-) | 965 | 0.38 |  |  | 62 | 0.31 |  | | 67 | | 0.25 |  | | 54 | | 0.26 |  | 47 | 0.50 |
| Smoking (+) or  excessive alcohol consumption (+) | 491 | 5.29 |  |  | 111 | 3.64 |  | | 172 | | 3.19 |  | | 167 | | 2.91 |  | 230 | 3.61 |
| **Colorectal cancer** |  |  |  |  |  |  |  | |  | |  |  | |  | |  |  |  |  |
| Smoking (-) and  excessive alcohol consumption (-) | 1,141 | 0.45 |  |  | 95 | 0.48 |  | | 144 | | 0.55 |  | | 100 | | 0.48 |  | 52 | 0.55 |
| Smoking (+) or  excessive alcohol consumption (+) | 290 | 3.12 |  |  | 74 | 2.43 |  | | 110 | | 2.04 |  | | 116 | | 2.02 |  | 145 | 2.27 |
| **Liver cancer** |  |  |  |  |  |  |  | |  | |  |  | |  | |  |  |  |  |
| Smoking (-) and  excessive alcohol consumption (-) | 758 | 0.30 |  |  | 52 | 0.26 |  | | 82 | | 0.31 |  | | 73 | | 0.35 |  | 33 | 0.35 |
| Smoking (+) or  excessive alcohol consumption (+) | 385 | 4.14 |  |  | 86 | 2.82 |  | | 134 | | 2.48 |  | | 150 | | 2.61 |  | 121 | 1.89 |

PYs indicates person-years.

Smoking (-): nonsmoking; (+): current smoking.

Excessive alcohol consumption (-): less than daily or <15g/d of pure alcohol consumption; (+): ≥15g/d of pure alcohol consumption.

STROBE Statement—checklist of items that should be included in reports of observational studies

|  | | Item No | Recommendation |
| --- | --- | --- | --- |
| **Title and abstract** | | 1 | (*a*) Indicate the study’s design with a commonly used term in the title or the abstract Page 1 line 1-3 |
|  |  |  | (*b*) Provide in the abstract an informative and balanced summary of what was done and what was found Page 3-4 |
| Introduction | | | |
| Background/rationale | | 2 | Explain the scientific background and rationale for the investigation being reported Page 6-7 |
| Objectives | | 3 | State specific objectives, including any prespecified hypotheses Page 7 line 14-20 |
| Methods | | | |
| Study design | | 4 | Present key elements of study design early in the paper Page 8 line 2-9 |
| Setting | | 5 | Describe the setting, locations, and relevant dates, including periods of recruitment, exposure, follow-up, and data collection Page 8 line 2-9 |
| Participants | | 6 | (*a*) *Cohort study*—Give the eligibility criteria, and the sources and methods of selection of participants. Describe methods of follow-up Page 8 line 3-5  *Case-control study*—Give the eligibility criteria, and the sources and methods of case ascertainment and control selection. Give the rationale for the choice of cases and controls NA  *Cross-sectional study*—Give the eligibility criteria, and the sources and methods of selection of participants NA |
|  |  |  | (*b*) *Cohort study*—For matched studies, give matching criteria and number of exposed and unexposed NA  *Case-control study*—For matched studies, give matching criteria and the number of controls per case NA |
| Variables | | 7 | Clearly define all outcomes, exposures, predictors, potential confounders, and effect modifiers. Give diagnostic criteria, if applicable Page 8-11 |
| Data sources/ measurement | | 8* | For each variable of interest, give sources of data and details of methods of assessment (measurement). Describe comparability of assessment methods if there is more than one group Page 8-11 |
| Bias | | 9 | Describe any efforts to address potential sources of bias Page 9-13 |
| Study size | | 10 | Explain how the study size was arrived at Page 8 line 10-18 |
| Quantitative variables | | 11 | Explain how quantitative variables were handled in the analyses. If applicable, describe which groupings were chosen and why Footnotes for all tables |
| Statistical methods | | 12 | (*a*) Describe all statistical methods, including those used to control for confounding Page 11-12 |
|  |  |  | (*b*) Describe any methods used to examine subgroups and interactions Page 12 line 12 – page 13 line 2 |
|  |  |  | (*c*) Explain how missing data were addressed Page 8 line 11 |
|  |  |  | (*d*) *Cohort study*—If applicable, explain how loss to follow-up was addressed Page 8 line 12  *Case-control study*—If applicable, explain how matching of cases and controls was addressed NA  *Cross-sectional study*—If applicable, describe analytical methods taking account of sampling strategy NA |
|  |  |  | (*e*) Describe any sensitivity analyses Page 13 line 3-7 |
| Results | | | |
| Participants | 13* | (a) Report numbers of individuals at each stage of study—eg numbers potentially eligible, examined for eligibility, confirmed eligible, included in the study, completing follow-up, and analysed Page 8 line 10-18 | |
|  |  | (b) Give reasons for non-participation at each stage Page 8 line 10-18 | |
|  |  | (c) Consider use of a flow diagram NA | |
| Descriptive data | 14* | (a) Give characteristics of study participants (eg demographic, clinical, social) and information on exposures and potential confounders Page 13 line 2-8, Table 1, Appendix table 1 | |
|  |  | (b) Indicate number of participants with missing data for each variable of interest Page 8 line 10 | |
|  |  | (c) *Cohort study*—Summarise follow-up time (eg, average and total amount) Page 13 line 11 | |
| Outcome data | 15* | *Cohort study*—Report numbers of outcome events or summary measures over time Page 13 line 22 – page 14 line 4 | |
|  |  | *Case-control study—*Report numbers in each exposure category, or summary measures of exposure NA | |
|  |  | *Cross-sectional study—*Report numbers of outcome events or summary measures NA | |
| Main results | 16 | (*a*) Give unadjusted estimates and, if applicable, confounder-adjusted estimates and their precision (eg, 95% confidence interval). Make clear which confounders were adjusted for and why they were included Table 2-3, footnotes for tables; Figure 1, legend (page 24); Appendix table 2-3, footnotes for tables | |
|  |  | (*b*) Report category boundaries when continuous variables were categorized Table 1-3, footnotes for tables; Appendix table 1-3, footnotes for tables | |
|  |  | (*c*) If relevant, consider translating estimates of relative risk into absolute risk for a meaningful time period NA | |
| Other analyses | 17 | Report other analyses done—eg analyses of subgroups and interactions, and sensitivity analyses Table 3, footnotes for tables; Figure 1, legend (page 25); Appendix table 2-4, footnotes for tables | |
| Discussion | | | |
| Key results | 18 | Summarise key results with reference to study objectives Page 16 line 5-11 | |
| Limitations | 19 | Discuss limitations of the study, taking into account sources of potential bias or imprecision. Discuss both direction and magnitude of any potential bias Page 19 line 16 – page 20 line 6 | |
| Interpretation | 20 | Give a cautious overall interpretation of results considering objectives, limitations, multiplicity of analyses, results from similar studies, and other relevant evidence Page 20 line 9-14 | |
| Generalisability | 21 | Discuss the generalisability (external validity) of the study results Page 20 line 2-6 | |
| Other information | | | |
| Funding | 22 | Give the source of funding and the role of the funders for the present study and, if applicable, for the original study on which the present article is based Page 4 | |

*Give information separately for cases and controls in case-control studies and, if applicable, for exposed and unexposed groups in cohort and cross-sectional studies.

**Note:** An Explanation and Elaboration article discusses each checklist item and gives methodological background and published examples of transparent reporting. The STROBE checklist is best used in conjunction with this article (freely available on the Web sites of PLoS Medicine at http://www.plosmedicine.org/, Annals of Internal Medicine at http://www.annals.org/, and Epidemiology at http://www.epidem.com/). Information on the STROBE Initiative is available at www.strobe-statement.org.
